# Congenital Arthrogryposis: An Extension of the 15q11.2 BP1-BP2 Microdeletion Syndrome?

**DOI:** 10.1155/2014/127258

**Published:** 2014-02-12

**Authors:** K. M. Usrey, C. A. Williams, M. Dasouki, L. C. Fairbrother, M. G. Butler

**Affiliations:** ^1^Departments of Psychiatry, Behavioral Sciences and Pediatrics, University of Kansas Medical Center, 3901 Rainbow Boulevard, MS 4015, Kansas City, KS 66160, USA; ^2^Division of Genetics and Metabolism, Department of Pediatrics, University of Florida College of Medicine, Gainesville, FL 32610, USA; ^3^Department of Neurology, University of Kansas Medical Center, Kansas City, KS 66160, USA; ^4^King Faisal Specialist Hospital and Research Center, Riyadh 11211, Saudi Arabia

## Abstract

The proximal 15q11–q13 region contains 5 breakpoints (BP1–BP5). The BP1-BP2 region spans approximately 500 kb and contains four evolutionarily conserved genes. The genes in this region are known to play a role in central nervous system development and/or function. Microdeletions within the 15q11.2 BP1-BP2 region have been reported in patients with neurological dysfunction, developmental delays, behavioral problems, and dysmorphic features. We report two unrelated subjects with the 15q11.2 BP1-BP2 microdeletion and presenting with congenital arthrogryposis, a feature which has not been previously reported as part of this newly recognized microdeletion syndrome. While arthrogryposis seen in these two subjects may be coincidental, we propose that congenital arthrogryposis may result from neurological dysfunction and involvement of the microdeletion of the 15q11.2 BP1-BP2 region, further expanding the phenotype of this microdeletion syndrome. We encourage others to report patients with this chromosome microdeletion and neurological findings to further characterize the clinical phenotype.

## 1. Introduction

The proximal 15q11–q13 region contains 5 breakpoints (BP1–BP5) located at low copy repeats implicated in causing chromosomal anomalies, primarily deletions, and duplications due to nonallelic homologous recombination [1, 2]. The most widely recognized deletion in this region is associated with two syndromes, Prader-Willi (PWS) and Angelman (AS), depending on the parent of origin and errors in genomic imprinting. The classical 15q11–q13 deletion seen in PWS or AS is of two types, a longer type I deletion involving BP1 and BP3 or the shorter type II deletion involving BP2 and BP3. Those with the longer type I deletion, in either PWS or AS, are reported with a more severe phenotype than those with the type II deletion [3–5].

The BP1-BP2 region spans approximately 500 kb and contains four evolutionarily conserved genes that are not imprinted: *NIPA1*, *NIPA2*, *CYFIP1*, and *TUBGCP5* [6]. These genes are implicated in playing a role in central nervous system development and function; for example, mutations of *NIPA1* are associated with spastic paraplegia [7, 8]. The related *NIPA2* gene is widely expressed in the central nervous system and encodes for a magnesium transporter [9]. The *CYFIP1* gene encodes a protein that interacts with FMRP, the protein product of the *FMR1* gene responsible for fragile X syndrome [10]. This syndrome is the most common cause of familial intellectual disability and primarily affects males [11]. The fourth gene, *TUBGCP5*, is a member of the cystoskeleton tubulin complex. Disturbed expression of these genes is reported in individuals with PWS; those with the longer type I deletion have lower expression than normal, while the genes are intact in those individuals carrying the smaller type II deletion [12].

Schizophrenia, autism, and speech delay have been reported in those with the 15q11.2 BP1-BP2 microdeletion [13–15]. Furthermore, Doornbos et al. [16] delineated the emerging phenotype of the 15q11.2 BP1-BP2 deletion to include developmental delay, behavioral abnormalities, motor apraxia, and dysmorphic features (ear abnormalities, cleft or narrow palate, and hypertelorism). In 2010, de Kovel et al. [17] reported an association between the 15q11.2 microdeletion and idiopathic generalized epilepsy. In 2011, Burnside et al. [18] reported their experience with approximately 17,000 cases referred for microarray analysis from 2008 to 2010. They found that 146 individuals carried microdeletions or duplications in the 15q11.2 BP1-BP2 region, which accounted for 0.86% of the total cases. Most individuals were under 18 years of age and many had neurological/behavioral impairment (e.g., 63%), speech problems (90%), developmental delays (59%), ataxia/coordination issues (30%), and/or hypotonia (20%). Recently, a report by Wong et al. in 2013 [19] suggested that the phenotype could be expanded to include tracheoesophageal fistula and congenital cataracts. Herein, we report two unrelated patients with the 15q11.2 BP1-BP2 microdeletion and congenital arthrogryposis.

## 2. Case Reports

*Case  1*. Our Caucasian female subject was born at full term and presented for genetic evaluation shortly after delivery due to bilateral hip dislocation with contractures, bilateral knee extension contractures, and bilateral equinovarus deformity. Limited wrist supination and finger flexions were noted at the metacarpophalangeal and proximal interphalangeal joints. The pregnancy history was unremarkable and the family history was negative for consanguinity, birth defects, or similarly affected individuals. Arthrogryposis multiplex congenita was diagnosed with no known cause.

**Figure 1:**
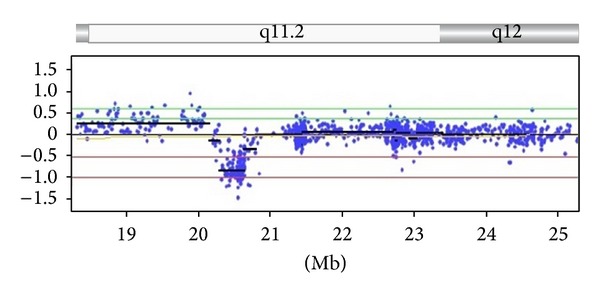
Chromosomal microarray analysis of Case 1 using the Combimatrix DNAarray Oligo 180 K (Combimatrix Diagnostics, Irvine, CA) showing the location of the deletion on chromosome 15 involving BP1-BP2 (20, 290, 386–20, 633, 303 bp from p terminus using content source from UCSC hg18 human genome (NCBI build 36, March 2006)). *Y* axis shows the chromosome copy number (0 = normal or nondeletion; −1.0 = deletion from a single chromosome). Genes in the deleted region are *CYFIP1*, *NIPA1*, *NIPA2*, and *TUBGCP5*.

At one month of age, she weighed 4.8 kg (90th centile) and her length was 54 cm (50th centile). On physical exam, she was found to be normocephalic with normal eye, skull, and ear appearance. She had moderate micrognathia. Bilateral hip dislocations and contractures were again noted with bilateral knee and ankle contractures and talipes equinovarus. The spine was straight. No oral lesions, facial clefts, or cardiac, abdominal, or skin abnormalities were noted. Laboratory evaluations included normal electrolytes, muscle and liver enzymes, and hematogram. Given the subject's age, no comment was made regarding developmental delays, but no seizures, hearing problems, or pathological reflexes were noted. No recognized genetic syndrome or cause of the arthrogryposis (muscle or neurological) was again identified. A chromosomal microarray analysis was obtained which showed a microdeletion at 15q11.2 BP1-BP2 (20, 290, 386–20, 633, 303 bp from the p terminus using UCSC hg18 version) (see Figure 1). Parental testing revealed the deletion to be paternally inherited, but the father had no obvious clinical abnormalities.

*Case  2*. Our Hispanic male subject presented for genetic evaluation at 11 years of age due to a history of severe arthrogryposis noted at birth and severe developmental disabilities. He was wheelchair bound with an adaptive resting device and had a tracheostomy with a G-tube in place. He was born to a 20-year-old G1P0 mother who had pregnancy induced hypertension but no other complications. There were no exposures to medications, alcohol, drugs, or tobacco. Intrauterine ultrasounds were reportedly normal. He was a spontaneous full term vaginal delivery with vacuum assistance. His birth weight was 3.2 kg (25th centile). Apgar scores were 8. He was an inpatient for 2 weeks after delivery due to hypotonia, seizure activity, and arthrogryposis. Perinatal investigations included an EEG which showed diffuse suppression of background cerebral activity with rare bifrontal sharp transients felt to be irritable cortical foci with potential for epileptic changes. A repeated EEG showed similar findings. At eight months of age, he sustained an apparent respiratory and cardiac arrest that may have contributed to his severe cognitive impairment.

At 11 years of age he weighed 27.0 kg (10th centile), his length was 133 cm (<10th centile), and his head circumference was 50 cm (2nd centile). No birth marks, seizures, or obvious craniofacial dysmorphism were noted. He did not establish eye contact. His hands were flexed with extended elbows in a typical arthrogrypotic position and the patient's behavior consisted of repeated midline stereotypic movements. Microcephaly, strabismus, and small feet, as well as quadriparesis with severe muscle atrophy of lower extremities, were noted on physical examination. No cardiac, pulmonary, genital, or renal anomalies were identified. Routine chromosome evaluation, two muscle biopsies done previously with no evidence of inflammatory problems or necrotic muscle fibers, and brain CT scan were interpreted as normal. A 15q11.2 BP1-BP2 microdeletion (20, 224, 751–20, 788, 605 bp from p terminus using UCSC hg18 version) was identified on chromosomal microarray analysis. Family history was negative for consanguinity, birth defects, or similarly affected individuals. Parental testing was not performed.

## 3. Discussion

Arthrogryposis has been reported in more than 300 disorders with several etiologies involving many causative genes. It is diagnosed in 1 in 3000 births with no known ethnic differences [20–22]. None of the four genes found in the 15q11.2 BP1-BP2 region have been reported to be associated with arthrogryposis. However, mutations in the *NIPA1* gene have been reported previously in autosomal dominant spastic paraplegia [7, 8]. Our first subject's father with the 15q11.2 BP1-BP2 microdeletion presented without clinical abnormalities, but unaffected family members with this chromosome finding have been previously reported [16, 18].

Congenital arthrogryposis could be related to neurological dysfunction associated with disturbed genes in this chromosome region and may be considered an extension of the microdeletion phenotype. Additionally, this phenotype may be due to a gene(s) disturbance or mutation present in the intact (nondeleted) alleles in this region. It may also be a coincidental finding or due to an unrelated defective gene elsewhere in the genome. The authors encourage the reporting of other individuals representing neurological deficits such as arthrogryposis to further expand the phenotype in this newly recognized microdeletion syndrome and to increase awareness of the clinical variation. Advanced genetic testing including exome sequencing of alleles in this region or elsewhere in the genome and noncoding RNA expression patterns impacting on genes in the 15q11.2 region should be undertaken and is currently underway in Case 1 to further delineate this interesting chromosome aberration and clinical variability.
